# A Rare Case of Erdheim-Chester Disease (Non-Langerhans Cell Histiocytosis) with Concurrent Langerhans Cell Histiocytosis: A Diagnostic and Therapeutic Challenge

**DOI:** 10.1155/2018/7865325

**Published:** 2018-05-16

**Authors:** Hamza Hashmi, Drew Murray, John Greenwell, Marwan Shaikh, Soumit Basu, Maxwell Krem

**Affiliations:** ^1^Division of Hematology and Oncology, University of Louisville, Louisville, KY, USA; ^2^Division of Pathology and Laboratory Medicine, University of Louisville, Louisville, KY, USA; ^3^Division of Hematology and Oncology, University of West Virginia, Morgantown, WV, USA; ^4^Geisinger Health System, Center for Blood and Marrow Transplant, Danville, PA, USA; ^5^Division of Blood and Marrow Transplant, University of Louisville, Louisville, KY, USA

## Abstract

Erdheim-Chester disease (ECD) is a rare non-Langerhans cell histiocyte disorder most commonly characterized by multifocal osteosclerotic lesions of the long bones demonstrating sheets of foamy histiocyte infiltrates on biopsy with or without histiocytic infiltration of extraskeletal tissues. ECD can be difficult to diagnose since it is a very rare disease that can affect many organ systems. Diagnosis is based on the pathologic evaluation of involved tissue interpreted within the clinical context. Patients who have the BRAF V600E mutation are treated first line with vemurafenib. For those without the mutation with symptomatic ECD, conventional or PEGylated interferon alpha is recommended. For patients who are either intolerant or nonresponsive to interferon alpha, systemic chemotherapy with or without corticosteroids can be used. We present a rare case of Erdheim-Chester disease with concurrent Langerhans cell histiocytosis which occurs in only one fifth of the cases and often presents as a diagnostic and therapeutic challenge.

## 1. Case Report

**Figure 1:**
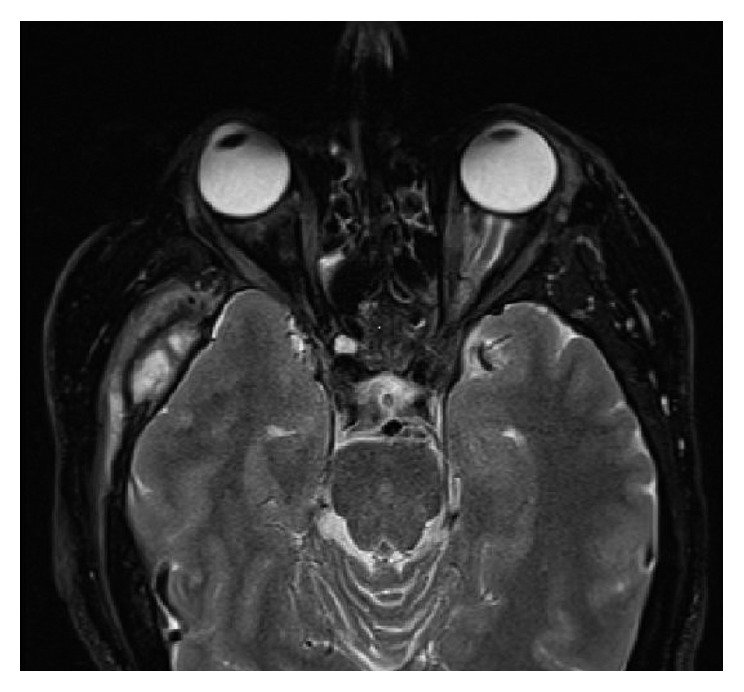
MRI brain showing a right orbital mass and a left mastoid mass.

We present the case of a 47-year-old Caucasian male who initially presented to the primary care physician 6 years ago with the chief complaint of left ear fullness of 2 weeks' duration. The patient also noticed that the left ear fullness was associated with tearing from the right eye. The patient was initially treated conservatively, with no subjective improvement in his symptoms. He underwent imaging studies which revealed a left mastoid/middle ear fossa mass and a right orbital mass (Figure 1). Imaging studies were followed by left mastoid-mass incisional biopsy and right orbital debulking. Biopsy of the mastoid/middle fossa area revealed Langerhans cell histiocytosis, and biopsy of the right eye orbit revealed non-Langerhans cell histiocytosis. Staging workup including imaging studies demonstrated a sizeable mass of soft tissue density encasing the infrarenal thoracoabdominal aorta and extending caudally into the proximal bilateral iliac arteries leading to right-sided hydroureter and hydronephrosis. Based on the radiographic appearance, this mass was consistent with Erdheim-Chester disease.

The surgical resection of the orbital mass was followed by six cycles of vinblastine 6 mg/m^2^ weekly and prednisone 40 mg/m^2^. Thereafter, he was started on monthly pulse prednisone and daily 6 mercaptopurine doses as maintenance therapy for 24 weeks.

**Figure 2:**
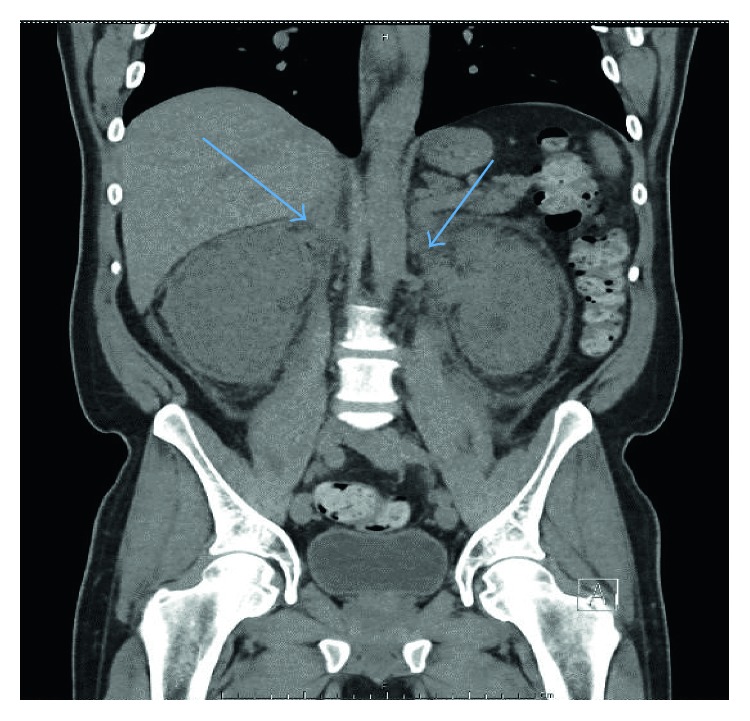
CT chest/abdomen/pelvis showing soft tissue encasing the adrenal glands, kidneys, infrarenal abdominal aorta, and soft tissue in the presacral space consistent with Erdheim-Chester disease.

The patient was relatively stable until about three years ago when he started experiencing diplopia in the right eye. CT scans revealed extensive bilateral intraconal enhancing soft tissue, infiltrative soft tissue involving the right sphenoid wing with extension into the pterygopalatine fossa and right temporal fossa, and enhancing soft tissue identified within bilateral frontal sinuses (Figure 2). Right orbitotomy and excisional biopsy of the orbital lesions confirmed relapse of non-Langerhans histiocytosis. Bone marrow biopsy demonstrated no evidence of histiocytic involvement.

**Figure 3:**
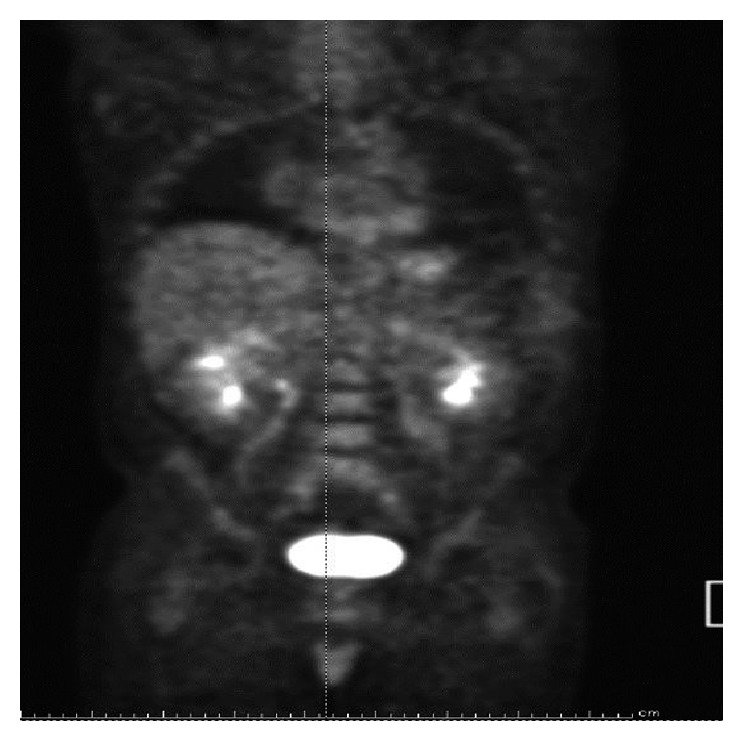
PET scan showing varying degrees of negligible, low-grade, and mild-to-moderate metabolic uptake consistent with multifocal soft tissue and osseous manifestations of Erdheim-Chester disease.

The patient was started on PEGylated interferon alpha to be given as 180 mcg subcutaneously weekly. Repeated CT evaluations over the next 3 years demonstrated no evidence of progression of disease on PEGylated interferon alpha-2a therapy (Figure 3).

**Figure 4:**
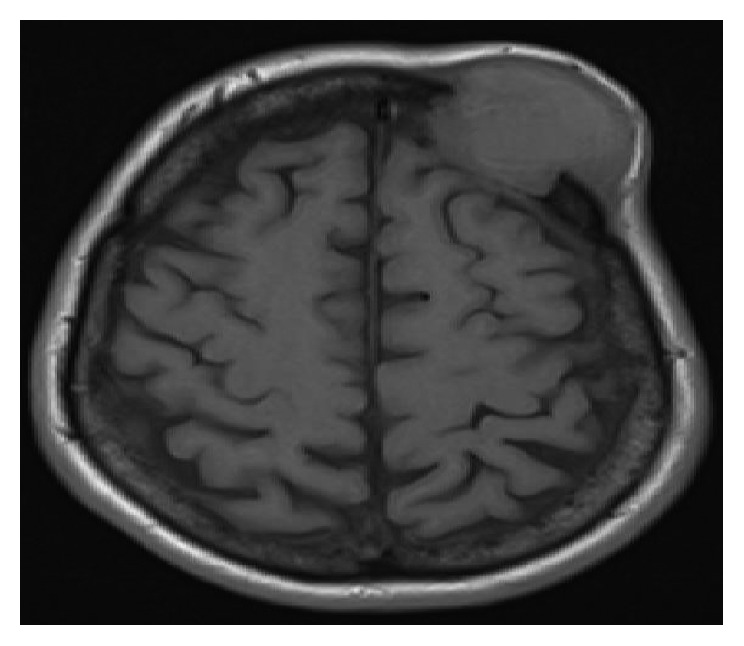
MRI brain showing calvarial mass lesion extending intracranially.

**Figure 5:**
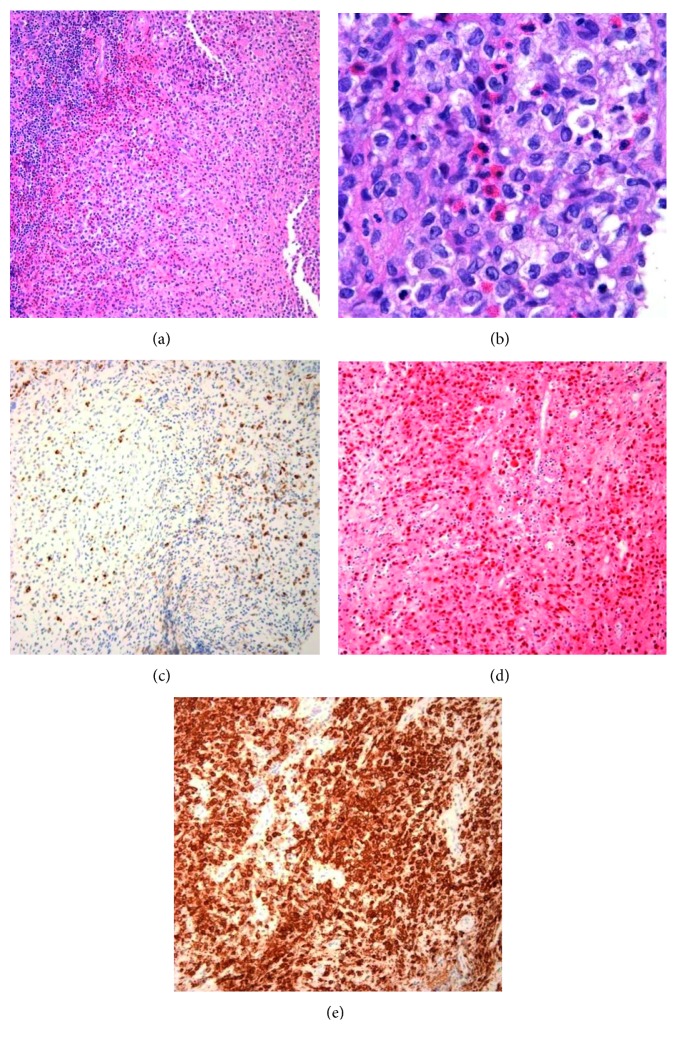
Representative sections of Langerhans cell histiocytosis. (a) Low-power (20x) image demonstrates a diffusive cellular infiltrate with prominent eosinophils. (b) High-power (100x) image demonstrates populations of eosinophils, macrophages with foamy cytoplasm, and large, variably elongated spindled cells with indented, grooved nuclei and eosinophilic granular cytoplasm. These cells are diffusely positive for (d) S100 and (e) CD1a and (c) focally positive for CD68, consistent with Langerhans cells.

About 6 months ago, the patient noticed a swelling on the frontal part of the scalp. MRI brain showed interval development of left calvarial mass lesion extending intracranially, associated with an epidural soft tissue component measuring 4 × 1.5 × 1.5 cm (Figure 4). The patient underwent resection of the mass with the pathology demonstrating both the skull and dural excisional specimens consistent with Langerhans cell histiocytosis with immunohistochemistry noting the cells as CD1a-positive, S100-positive, and largely CD68-negative (Figure 5). BRAF mutation was not tested on the biopsy specimen. As a result of this recurrence of Langerhans cell histiocytosis, he underwent PET-CT restaging that showed a stable disease from the prior studies. The patient underwent local radiation to the scalp lesion to consolidate the resection. Besides the use of corrective lens for diplopia, he continues to do fairly well and is largely symptom free on weekly interferon therapy.

## 2. Discussion

### 2.1. Epidemiology and Pathophysiology

Erdheim-Chester disease (ECD) is a rare form of non-Langerhans cells histiocytosis. Approximately 500 cases have been reported in the literature with far fewer cases occurring concurrently with Langerhans cell histiocytosis (LCH) [1]. However, 20% of patients diagnosed with ECD have or will develop mixed disease (concurrent LCH and ECD) [2]. ECD has been reported in all age populations, but most frequently presents in the fifth decade of life with a male prevalence of 3 : 1 [3, 4].

Recent studies have identified a high prevalence of the BRAF V600E mutation in patients with ECD. This gene is known to participate in oncogene-induced senescence [5]. A 2014 trial compared patients with mixed histiocytosis (concurrent LCH and ECD) and identified 48% of the patients were diagnosed simultaneously, while the remaining 52% had a diagnosis of LCH preceding the ECD [2]. Also, 82% of patients with ECD and 69% of those with LCH possessed BRAF V600E mutations [2]. This same oncogene has been identified in ECD and LCH, but not in other histiocytic proliferative disorders suggesting that it is a catalyst for pathology [2]. Other less common mutations such as MAP2K1, N/KRAS, ARAF, PIK3CA, and ERBB3 have also been noted in LCH and ECD [6]. These common genes have prompted reclassification from the separate entities of Langerhans cell histiocytosis and non-Langerhans cell histiocytosis among histiocytosis and macrophage-related neoplasms to include ECD and LCH in a “Langerhans subtype” category [6].

### 2.2. Clinical Manifestations

The most common clinical manifestations of ECD depend on the site of involvement. Literature review of 259 patients diagnosed with biopsy-proven ECD identified the most common presentations including bone pain (26%), neurologic features (23%), diabetes insipidus (22%), and constitutional symptoms (20%) [3]. Another study noted exophthalmos in 20–30% of patients along with xanthelasmas as the most common cutaneous manifestation [6]. Typically, initial workup reveals skeletal involvement with at least one nonbone involved site [3]. A retrospective case study showed that the long bones were the most commonly involved site (95%) followed by the maxillary sinus (59%), large blood vessels (59%), retroperitoneum (59%), heart (57%), lungs (46%), central nervous system (CNS) (41%), skin (27%), orbits (22%), and pituitary gland (22%) [7, 8].

Prognosis is generally poor, and no definitive cure for the disease is currently available. Cardiovascular and CNS involvement is usually associated with the worst prognosis [9]. Retrospective case series of patients treated with the conventional treatment of interferon alpha therapy have noted 1- and 5-year survival rates of 96% and 68%, respectively [10]. Although no updated data are available at this time, based on the overall response rate to newly approved BRAF inhibitor vemurafenib, prognosis is likely to improve [11].

### 2.3. Diagnostic Workup

**Figure 6:**
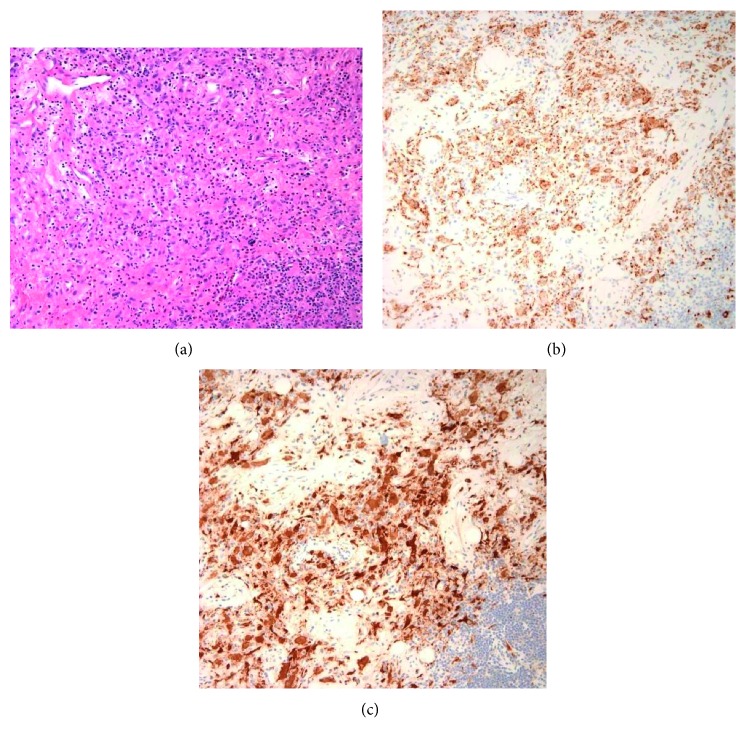
Non-Langerhans cell histiocytosis: (a) low-power (20x) image shows a histiocytic proliferation which is diffusely positive for CD68 and resembles that of Langerhans cell histiocytosis but with a comparatively low amount of eosinophils and negative immunostaining for CD1a. (b) CD68+. (c) Factor XIII+.

Definitive diagnosis of ECD requires biopsy identifying infiltration of tissue by foamy mononucleated histiocytes with small nuclei. Rare multinucleated histiocytes known as Touton cells are also observed [6]. Mild fibrosis is typically present and increases with severity. Birbeck granules typical of Langerhans cell histiocytosis are absent in ECD. Multiple biopsies are typically obtained from cutaneous lesions due to the ease of access and sufficient histopathological yield [4]. If obtained, biopsies of osteosclerotic bone lesions should contain enough tissue to allow for genetic testing for the presence of previously discussed genetic mutations as well as decalcification for histopathology [4]. Cellular markers identified by immunohistochemistry of ECD cells are CD68 and CD163, while markers CD1a and CD207 of S100 protein are usually negative [4, 9] (Figure 6). Histopathologic examination differentiates ECD from other forms of histiocytosis and macrophage-related proliferative disorders such as LCH, juvenile xanthogranuloma, Rosai-Dorfman disease, and hemophagocytic lymphohistiocytosis [6].

Prior to establishing treatment plans, patients require staging and genetic studies. Typical imaging consists of an MRI of the brain, an MRI or CT scan of the entire aorta, a cardiac MRI, a transthoracic echocardiogram, and a fluoro-d-glucose PET scan or CT scan of the chest, abdomen, and pelvis [4]. Radiologic findings of symmetric diaphyseal and metaphyseal otosclerosis detected by radiotracer uptake on bone scan or PET were detected in 96% of patients diagnosed with ECD. Other common findings are dense perinephric fat in 68% of cases and periaortic sheathing in 66% [4]. Based on patient presentation, additional studies to help identify the extent of the disease include MRI of the orbits, renal artery ultrasound, high-resolution CT of the chest, pulmonary function tests, testicular ultrasound, and electromyography [4].

### 2.4. Treatment and Surveillance

In the rare event of ECD diagnosis with asymptomatic disease with no objective evidence of CNS involvement or organ dysfunction, delaying treatment with adequate screening is reasonable [4]. In patients with BRAF V600E mutations, treatment should be initiated with newly FDA-approved agent vemurafenib. In patients without this mutation, interferon alpha is the therapeutic regimen with the most supportive evidence. Other treatment regimens including anticytokine-directed treatment (anakinra, infliximab, and tocilizumab), systemic chemotherapy, glucocorticoids, and palliative radiation have been shown to be efficacious [4].

Patients who carry BRAF V600E mutation should be considered for BRAF inhibitor therapy. Vemurafenib is a serine/threonine kinase inhibitor of activating point mutations in BRAF and was FDA approved for treatment of ECD on November 6, 2017 [12]. Estimations of 82% BRAF mutation prevalence in ECD make this agent a good alternative therapy [2]. The VE BASKET study observed 22 adult patients who received 960 mg BID initially with dose reductions to 720 mg BID (8 patients) and 480 mg BID (14 patients) [11]. Partial response was observed in 54% of patients with one patient achieving complete remission [11]. Serious adverse effects included joint pain, fatigue, arrhythmias fatigue, and hair loss. Recommended dose is 960 mg daily until intolerable toxicity or disease progression. Dose may be reduced to 720 mg BID or 480 mg BID in case significant toxicity develops. Toxicity is monitored with physical exam, ECG, liver function tests, ocular exams, and basic metabolic profile. This drug has also been shown to increase the prevalence of new primary cutaneous cancers, squamous cell carcinomas, noncutaneous malignancies, and tumor promotion of BRAF wild-type melanomas.

Previously regarded as first-line treatment, interferon alpha or PEGylated interferon alpha has the most robust supporting evidence for survival benefit in ECD [13]. The largest study was an observational cohort study of 53 patients treated with interferon alpha, which revealed improved overall survival compared to other therapies, and INF-a treatment independently improved survival in a multivariate analysis [10]. Optimal dosing is not established, but case studies have shown that severe disease (CNS or cardiac involvement) responds to high doses (9 mIU 3 times per week of INF-a) [14]. Optimal duration of treatment is unclear; some patients may receive therapy for up to 3 years [4]. Potential toxicities of interferon alpha include typical constitutional symptoms, neuropsychiatric symptoms, gastrointestinal symptoms, alopecia, pruritus, transaminitis, and myelosuppression [15]. Many practitioners favor the use of PEGylated INF-a due to its preferable adverse effect profile and convenience of dosing [15].

For patients who are either intolerant or nonresponsive to interferon alpha therapy, alternative treatment options include systemic chemotherapy with or without corticosteroids. Typical regimens for LCH and theoretical treatment for ECD is a 24-week treatment plan comprising weekly vinblastine and etoposide for six weeks followed by vinblastine and etoposide every three weeks for the remaining duration of the treatment course [16]. Use of other chemotherapeutic agents such as vinca alkaloids, anthracyclines, cyclophosphamide, methotrexate, cladribine, and leucovorin has been reported in the form of case reports [4, 17]. Although corticosteroids have not demonstrated any survival benefit, their use has shown symptomatic benefits with the sequelae of disease such as exophthalmos and side effects of chemotherapy [10]. The use of radiotherapy is considered palliative since it is practically ineffective, and recurrences are common [18].

Due to the rarity of the condition, only a limited number of drugs from clinical trials have become FDA-approved treatment regimens. Small case studies have noted the efficacy of anticytokine-directed therapy using agents such as anakinra (Il1 receptor antagonist), canakinumab (Il1 receptor antagonist), and infliximab (anti-TNF) [19–22]. Tocilizumab, an IL6 receptor antagonist, is currently in a phase 2 clinical trial (NCT01727206) for the treatment of ECD. Imatinib, a tyrosine kinase inhibitor, has shown some improvements in one-third of patients with platelet-derived growth factor receptor beta (PDGFR-b) mutation; however, two-thirds of the patients (especially with CNS involvement) experienced deterioration [23]. Autologous bone marrow transplant has been utilized with success in a small number of refractory cases of histiocytic disorders including two patients with ECD [1, 24].

Disease monitoring is typically done at 3- to 6-month intervals with flouro-d-glucose PET scans. Once the disease stabilizes, PET scans can be done less frequently as per clinician discretion [4]. As no biomarkers exist to monitor treatment, monitoring C-reactive protein levels has been found useful [25]. With a lack of consensus guidelines regarding treatment strategies, the duration of treatment is also debatable. Indefinite treatment is preferred, but given the existence of cases with minimal disease or prolonged intervals without disease progression, treatment cessation is feasible [4].

## 3. Conclusion

Erdheim-Chester disease (ECD) is a rare non-Langerhans histiocyte disorder, most commonly characterized by multifocal osteosclerotic lesions of the long bones demonstrating sheets of foamy histiocyte infiltrates on biopsy with or without histiocytic infiltration of extraskeletal tissues. Since it is a very rare disease that can affect many organ systems, diagnosis can be challenging. Diagnosis is based on the pathologic evaluation of involved tissue interpreted within the clinical context. Not all patients with ECD require treatment at the time of diagnosis. Patients who are asymptomatic without evidence of central nervous system involvement or organ dysfunction can simply be observed. With vemurafenib becoming the first FDA-approved drug for the treatment of ECD, patients who possess BRAF V600E mutation should be considered for BRAF inhibitor therapy. In patients without the mutation or those who are intolerant to the drug, conventional or PEGylated interferon alpha is the recommended treatment. Further treatment options include the use of systemic chemotherapy with or without corticosteroids.
